# Adult-onset giant cell glioblastoma with biallelic PMS2 inactivation in Lynch syndrome

**DOI:** 10.1093/noajnl/vdag256

**Published:** 2026-10-22

**Authors:** Muhammed Onel, Luke Gingell, Aysegul Kasay, Mohammad Muhsin Chisti

**Affiliations:** Internal Medicine, Corewell Health William Beaumont University Hospital, Royal Oak, Michigan, USA; Internal Medicine, Corewell Health William Beaumont University Hospital, Royal Oak, Michigan, USA; Independent Researcher, Troy, Michigan, USA; Hematology & Oncology, Corewell Health William Beaumont University Hospital, Royal Oak, Michigan, USA

**Keywords:** giant cell glioblastoma, immune checkpoint inhibition, Lynch syndrome, mismatch repair deficiency, PMS2

## Abstract

Mismatch repair (MMR)-deficient glioblastomas represent a rare molecular subset with important hereditary and therapeutic implications. We report a 58-year-old man with adult-onset giant cell glioblastoma showing isolated PMS2 loss, microsatellite instability-high status, ultra-high tumor mutational burden, and confirmed biallelic PMS2 inactivation from a likely pathogenic germline PMS2 splice-site variant and somatic PMS2 nonsense mutation, revealing previously unrecognized Lynch syndrome. DNA methylation profiling classified the tumor as a high-grade glioma with MMR deficiency, with a methylation match to diffuse high-grade glioma, IDH-wildtype, subtype E. Given O⁶-methylguanine-DNA methyltransferase (MGMT) promoter unmethylation and MMR deficiency, temozolomide was omitted, and the patient received adjuvant radiation therapy with concurrent and maintenance pembrolizumab. Surveillance MRI at 5 months after gross total resection showed no evidence of recurrence. This case highlights giant cell morphology and ultra-high tumor mutational burden as potential clues to underlying MMR deficiency and supports consideration of reflex MMR immunohistochemistry, germline evaluation, and immune checkpoint inhibition in selected dMMR/MSI-H glioblastomas.

Glioblastoma (GB) is the most common primary malignant brain tumor in adults, with a median survival of approximately 15 months despite standard chemoradiation.^1^^,^^2^ Mismatch repair deficiency (dMMR) defines a rare subset of GB with distinct therapeutic implications.^3^^,^^4^ Biallelic MMR gene inactivation with somatic hypermutation was identified in approximately 2% of consecutive primary treatment-naïve IDH-wildtype GBs in a prospective adult series.^5^ Among hereditary causes, Lynch syndrome predisposes to brain tumors as a rare manifestation, with most Lynch-associated gliomas linked to MSH2 or MLH1; PMS2-associated gliomas in adults are exceptionally uncommon.^3^^,^^6^ Giant cell GB is a rare histologic variant linked to underlying dMMR.^7^ Adult-onset giant cell GB arising in the setting of PMS2-associated Lynch syndrome, to our knowledge, has not been reported in the literature. We report a 58-year-old man with giant cell GB demonstrating isolated PMS2 loss and confirmed biallelic inactivation resulting from a germline splice-site variant and a somatic nonsense mutation.

## Case Presentation

### Hospital Course

A 58-year-old man with no significant past medical history and a family history notable only for prostate cancer in his father presented to the hospital with a mild headache, confusion, and behavioral changes. Noncontrast CT of the head in the emergency department revealed a large cystic-solid mass in the left frontal lobe with prominent mass effect. Brain MRI confirmed a large cystic-solid mass in the left frontal lobe with significant mass effect, 10 mm of rightward midline shift, and mild perilesional edema, without additional intracranial lesions. Postoperative MRI confirmed gross total resection of the enhancing tumor component and showed expected postsurgical changes (Figure 1). The patient was discharged in stable condition, with cancer treatment planning deferred to the outpatient setting.

**Figure 1.**
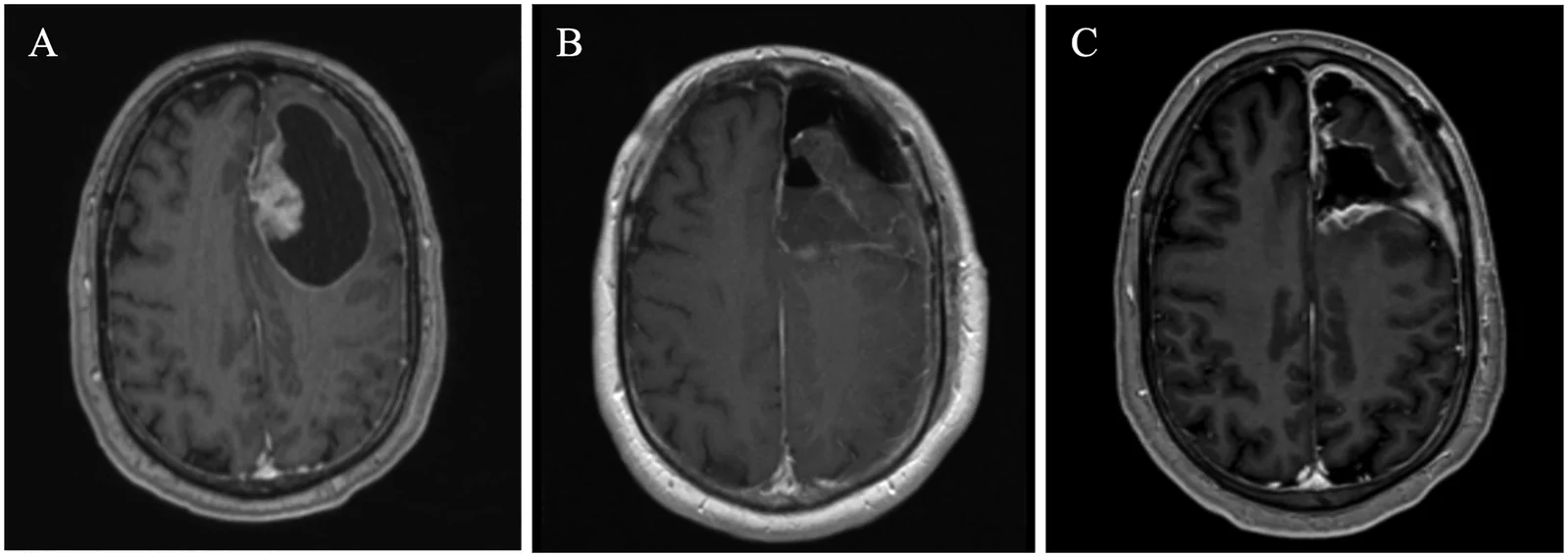
Serial brain MRI (axial postcontrast T1-weighted images). (A) Preoperative MRI demonstrating a large cystic-solid mass in the left frontal lobe with an enhancing solid nodular component measuring 4.1 × 1.5 × 3.8 cm, associated with 10 mm of rightward midline shift. (B) Immediate postoperative MRI confirming gross total resection of the enhancing tumor component with expected postsurgical changes. (C) Surveillance MRI at 5 months postresection following completion of adjuvant radiation therapy showing no suspicious foci of enhancement surrounding the resection bed.

### Histopathology and Immunohistochemistry

Histopathologic examination revealed a markedly hypercellular neoplasm composed of pleomorphic astrocytic cells with numerous enlarged, multinucleated giant cells exhibiting marked nuclear atypia. Focal areas of necrosis with degenerative changes, incipient microvascular proliferation, and infiltration into surrounding brain parenchyma were identified. Neurofilament immunostaining demonstrated extensive tumor infiltration into reactive brain tissue. Based on these findings, the favored diagnosis was GB with giant cell features.

### Molecular and Genetic Profiling

Immunohistochemistry for IDH1 R132H was negative, ATRX expression was lost, and p53 demonstrated diffuse nuclear positivity in over 40% of tumor cells. The Ki-67 proliferation index exceeded 40%. Institutional next-generation sequencing (NGS) (50-gene panel) confirmed the absence of IDH1/IDH2 mutations, consistent with IDH-wildtype GB, and identified pathogenic ATRX and TP53 alterations. MGMT promoter methylation was not detected.

Mismatch repair (MMR) protein immunohistochemistry demonstrated isolated loss of PMS2 expression with retained MLH1, MSH2, and MSH6, indicating dMMR. Institutional NGS identified 2 somatic PMS2 nonsense variants: the clonal p.R315* (c.943C > T; variant allele frequency [VAF] 44%) and likely subclonal p.R211* (c.631C > T; VAF 15%). The same platform reported MSI status as borderline (18%) and a tumor mutational burden (TMB) of 317 mutations/Mb. Caris comprehensive genomic profiling (Caris CGP; 228-gene panel) demonstrated microsatellite instability-high (MSI-H) status, tumor mutational burden-high (TMB-H) at 139 mutations/Mb, and independently detected the clonal p.R315* variant; p.R211* variant was not detected, likely falling below the platform’s reporting threshold. A pathogenic POLE variant (p.S461P, c.1381T > C) was also identified. Caris CGP copy-number profiling demonstrated retention of chromosome 9p without evidence of arm-level loss, with no CDKN2A/CDKN2B homozygous deletion. The borderline MSI result and differing TMB estimates between platforms may reflect differences in panel size, genomic coverage, and bioinformatic methods; both platforms nevertheless demonstrated a markedly elevated TMB.

Genome-wide DNA methylation profiling performed through the National Cancer Institute classified the tumor as high-grade glioma with dMMR, with a methylation match to diffuse high-grade glioma, IDH-wildtype, subtype E.

The isolated loss of PMS2 expression on immunohistochemistry raised concern for an underlying germline MMR defect, prompting hereditary cancer evaluation. Germline testing identified a likely pathogenic heterozygous PMS2 splice-site variant (c.804-10T > G), confirming Lynch syndrome. The germline splice-site variant is presumed to inactivate one PMS2 allele, while the clonal somatic p.R315* nonsense mutation represents the second hit on the remaining wild-type allele, confirming biallelic PMS2 inactivation. The patient was referred to genetic counseling for Lynch syndrome education, discussion of cancer surveillance recommendations, and coordination of cascade testing for at-risk family members.

The molecular, genomic, and germline findings are summarized in Table 1.

### Treatment

Given MGMT promoter unmethylation and dMMR with MSI-H/TMB-H status, temozolomide was omitted. The patient underwent adjuvant partial-brain radiation therapy with 60 Gy in 30 fractions. Pembrolizumab 200 mg intravenously every 3 weeks was initiated during the radiation course and continued following completion of radiation therapy.

### Follow-Up

At 5 months following gross total resection, after completion of adjuvant radiation therapy and 4 months from pembrolizumab initiation, surveillance brain MRI demonstrated no evidence of recurrence.

The patient has completed 6 cycles of pembrolizumab with only grade 1 arthralgia and myalgia, maintaining a Karnofsky Performance Status of 90. Ongoing surveillance follows the immunotherapy Response Assessment in Neuro-Oncology (iRANO) criteria given immune checkpoint inhibition.

## Discussion

This case demonstrates adult-onset GB arising from heterozygous PMS2-associated Lynch syndrome with somatic inactivation of the remaining PMS2 allele. This mechanism of biallelic inactivation is more commonly reported for MSH2 and MLH1 in Lynch syndrome-associated gliomas (80% in one multi-cohort study), whereas PMS2 variants were rare and typically associated with constitutional dMMR in childhood rather than heterozygous Lynch syndrome in adults.^3^ The patient’s presentation at age 58 is notable given the declining prevalence of dMMR with age in IDH-wildtype GB, reported at 12.5% in patients aged less than 40 years, 2.6% in those aged 40–49 years, and 1.6% in those aged 50 years and older.^4^ Nevertheless, Suwala et al.^8^ demonstrated that primary MMR-deficient gliomas can occur in elderly patients. Because heterozygous PMS2 variants have relatively low penetrance for extracolonic malignancies, the hereditary nature of the syndrome may remain clinically occult, particularly in older adults without a strong family history.^6^ The tumor’s giant cell morphology was an important clue to the underlying biology. Multinucleated giant cells have been associated with dMMR in up to 77% of dMMR gliomas in one series, suggesting a link with genomic instability.^9^ This is also supported by the NCCN CNS guidelines, which note that tumors with de novo replication repair deficiency “often have features of the giant cell variant of GB.”^7^ The molecular profile of this tumor, including TP53 mutation, ATRX loss, and absence of EGFR amplification, is consistent with the characteristic genomic landscape of giant cell GB and overlaps with the molecular features enriched in primary dMMR gliomas.^3^^,^^10^

The convergence of giant cell morphology, isolated PMS2 loss with confirmed biallelic PMS2 inactivation, MSI-H status, and ultra-high TMB provides a coherent clinicopathologic pattern. Loss of MMR fidelity through germline and somatic PMS2 inactivation likely drove the extreme mutational burden and may have contributed to the giant cell morphology. Although causality cannot be established from a single case, this constellation may serve as a recognizable signature warranting comprehensive molecular profiling and germline evaluation. DNA methylation profiling classified the tumor as high-grade glioma with dMMR, matching diffuse high-grade glioma, IDH-wildtype, subtype E. This aligns with the epigenetic heterogeneity reported by Hadad et al.,^5^ in which most de novo replication repair-deficient GBs matched diffuse pediatric-type high-grade glioma, RTK1 subclass A, while one matched subtype E and 2 did not align with any established reference class. These findings align with Suwala et al.^8^ who proposed primary MMR-deficient glioma as a distinct type of IDH-wildtype glioma, supporting the emerging view that these tumors may represent a biologically distinct entity rather than a variant of conventional GB.

These findings suggest that giant cell features in GB should prompt MMR immunohistochemistry regardless of age or family history and that MMR loss warrants paired tumor and germline evaluation to distinguish sporadic deficiency from Lynch syndrome and enable cascade testing. Additionally, 2 somatic BRCA2 frameshift variants were identified but likely represent passenger events in an ultra-hypermutated context.

Temozolomide is considered the cornerstone chemotherapy for GB.^2^ However, its cytotoxicity depends on intact MMR function; even modest reductions in MMR protein expression confer resistance.^11^ In this patient, MGMT promoter unmethylation further reduced the expected benefit of alkylating chemotherapy.^1^ Together, these factors supported omission of temozolomide.

The rationale for pembrolizumab was based on both tumor-agnostic evidence and glioma-specific biologic considerations. Pembrolizumab has FDA approval for MSI-H/dMMR solid tumors, which generate abundant neoantigens capable of eliciting antitumor responses.^12^

Glioma-specific evidence, however, remains mixed. Bouffet et al.^13^ reported durable responses to nivolumab in 2 siblings with recurrent multifocal biallelic MMR-deficient GB and ultra-hypermutated tumors with secondary polymerase mutations. Negm et al.^3^ similarly found that immune checkpoint blockade was associated with improved survival compared with conventional chemoradiotherapy in primary dMMR gliomas. In contrast, a prospective pilot study of pembrolizumab in recurrent high-grade gliomas with MMR protein loss showed no objective responses, although the TMB in that cohort was substantially lower than in the present case.^14^ Fernandez et al. helped reconcile these discordant findings by identifying biologically distinct dMMR glioma subgroups. Tumors with secondary mutations in replicative DNA polymerases (POLE/POLD1) appear ultra-hypermutated, immune-hot, and more responsive to immunotherapy, whereas IDH1-mutant dMMR gliomas are generally immune-cold and resistant.^15^ Notably, Caris CGP identified a pathogenic POLE variant (p.S461P), consistent with secondary polymerase proofreading deficiency. This finding, together with the ultra-high TMB, places this tumor within the ultra-hypermutated, immune-hot subgroup identified by Fernandez et al. as most likely to respond to immune checkpoint inhibition. In a prospective cohort of 9 de novo replication repair deficient GBs, 5 patients received immune checkpoint blockade and 4 survived beyond 3 years; median overall survival for the entire 9-patient cohort was 36.8 months versus 15.5 months for 450 conventional GBs (*P* < 0.001).^5^ Suwala et al.^8^ further reported improved overall survival in primary MMR-deficient gliomas with TMB >50 mutations/Mb and balanced 9p status; the present tumor meets both criteria. The decision to initiate pembrolizumab concurrently with radiation therapy was supported by preclinical and emerging clinical evidence suggesting that radiation may enhance antitumor immunity and synergize with immune checkpoint blockade. However, prospective data specifically evaluating concurrent radiation and immunotherapy in primary dMMR gliomas remain limited.

Surveillance imaging showed no evidence of recurrence. Because immune checkpoint inhibition can cause pseudoprogression, radiographic changes must be interpreted cautiously. The iRANO criteria recommend confirming suspected progression on follow-up imaging at least 3 months later unless there is clear clinical deterioration.^16^

### Limitations

As a single case, causal inferences regarding treatment efficacy cannot be drawn. PD-L1 was not assessed, though the rationale for pembrolizumab rested on the tumor-agnostic MSI-H/dMMR indication. Follow-up is early, and concurrent radiation and pembrolizumab preclude attributing the favorable imaging to either modality alone.

## Conclusion

This case of giant cell GB with biallelic PMS2 inactivation and previously unrecognized Lynch syndrome highlights several actionable clinical implications. First, MMR immunohistochemistry should be considered in GBs with giant cell morphology or unexpectedly high TMB, regardless of patient age or family history. Second, loss of any MMR protein in a treatment-naïve GB should prompt molecular testing and germline evaluation, as paired analysis may reveal previously unrecognized carriers and enable cascade testing for at-risk relatives. Third, dMMR/MSI-H GBs with ultra-high TMB and secondary polymerase mutations, including POLE, may represent biologically favorable candidates for immune checkpoint inhibition, although prospective validation is needed.

**Table 1.** Molecular and genomic profile of the glioblastoma

Test	Method	Result	Clinical significance
IDH1 R132H	IHC	Negative	Confirms IDH-wildtype GB
IDH1/IDH2 sequencing	Institutional NGS	No mutations detected	Confirms IDH-wildtype GB
ATRX	IHC; Institutional NGS and Caris CGP	Loss of expression; p.G1934Rfs*8 and p.L359Tfs*3 (both pathogenic)	Consistent with giant cell GB molecular profile
p53	IHC	Diffuse positivity (>40%)	Consistent with missense TP53 mutation
TP53	Institutional NGS; Caris CGP	p.N239D (pathogenic); p.N239D (pathogenic), p.L111M (likely pathogenic)	Consistent with giant cell GB
MGMT promoter	Methylation-specific assay	Unmethylated	Reduced benefit from temozolomide
Ki-67	IHC	>40%	High proliferative index
MMR panel	IHC	Isolated PMS2 loss; MLH1, MSH2, MSH6 retained	dMMR
MSI status	Institutional NGS; Caris CGP	Borderline; MSI-H	Eligible for pembrolizumab
TMB	Institutional NGS; Caris CGP	317 mutations/Mb; 139 mutations/Mb	TMB-H by both platforms
DNA methylation profiling	Genome-wide DNA methylation array (NCI)	High-grade glioma with mismatch repair deficiency; methylation match to diffuse high-grade glioma, IDH-wildtype, subtype E	Supports dMMR high-grade glioma
Somatic PMS2	Institutional NGS; Caris CGP	p.R315* (pathogenic, VAF 44%, clonal) and p.R211* (pathogenic, VAF 15%, likely subclonal); p.R315* (pathogenic)	Clonal p.R315* represents second hit confirming biallelic inactivation; subclonal p.R211 likely consequence of hypermutator phenotype
POLE	Caris CGP	p.S461P (pathogenic)	Secondary polymerase proofreading deficiency; supports ultra-hypermutated, immune-hot subgroup
BRCA2	Caris CGP	p.W1692fs and p.N830fs (both pathogenic, somatic)	Somatic DNA repair pathway alteration; may represent passenger events in hypermutated context
BRAF	Caris CGP	No mutation, no fusion	Supports GB over PXA
EGFR	Caris CGP	No amplification, no fusion, no EGFRvIII	Consistent with giant cell GB
NTRK1/2/3	Caris CGP	No fusion detected	Not eligible for TRK inhibitor
H3F3A/H3F3B/HIST1H3B	Caris CGP	No mutation detected	Excludes histone-mutant glioma
CDKN2A/CDKN2B	Caris CGP	No deletion, no mutation	Relevant for glioma classification
PPM1D	Caris CGP	No mutation detected	Excludes treatment-associated hypermutation; supports de novo hypermutated phenotype
Germline PMS2	Germline panel testing	c.804-10T > G (likely pathogenic, splice-site)	Confirms Lynch syndrome

Summary of immunohistochemical, molecular, and germline testing results with corresponding clinical significance. For rows with multiple testing methods separated by semicolons, results are listed in the corresponding order. Institutional NGS (50-gene panel) and comprehensive genomic profiling (Caris Life Sciences; 228-gene panel) were performed on tumor tissue. Genome-wide DNA methylation profiling was performed at the National Cancer Institute. Germline testing was performed on peripheral blood. Variants are reported using HGVS protein nomenclature;

* denotes a stop codon (nonsense variant) and “fs” denotes a frameshift variant. Differences in TMB and MSI results between platforms reflect variation in panel size and bioinformatic algorithms. Variant allele frequencies (VAFs) are reported from institutional NGS. Full details are available from the corresponding authors upon request.

Abbreviations: CGP, comprehensive genomic profiling; dMMR, mismatch repair deficiency; EGFR, epidermal growth factor receptor; GB, glioblastoma; HGVS, Human Genome Variation Society; IHC, immunohistochemistry; MMR, mismatch repair; MSI, microsatellite instability; MSI-H, microsatellite instability-high; NCI, National Cancer Institute; NGS, next-generation sequencing; PXA, pleomorphic xanthoastrocytoma; TMB, tumor mutational burden; TMB-H, tumor mutational burden-high; TRK, tropomyosin receptor kinase.

## Data Availability

The data underlying this article are available in the article itself. Additional data will be shared on reasonable request to the corresponding authors.
